# Maize stalk stiffness and strength are primarily determined by morphological factors

**DOI:** 10.1038/s41598-021-04114-w

**Published:** 2022-01-14

**Authors:** Christopher J. Stubbs, Ryan Larson, Douglas D. Cook

**Affiliations:** 1 School of Computer Sciences and Engineering, Fairleigh Dickinson University, Teaneck, NJ 07666 USA; 2 Department of Mechanical Engineering, Brigham Young University, Provo, UT 84602 USA

**Keywords:** Mechanical engineering, Plant sciences

## Abstract

The maize (Zea mays) stem is a biological structure that must balance both biotic and structural load bearing duties. These competing requirements are particularly relevant in the design of new bioenergy crops. Although increased stem digestibility is typically associated with a lower structural strength and higher propensity for lodging, with the right balance between structural and biological activities it may be possible to design crops that are high-yielding *and* have digestible biomass. This study investigates the hypothesis that geometric factors are much more influential in determining structural strength than tissue properties. To study these influences, both physical and in silico experiments were used. First, maize stems were tested in three-point bending. Specimen-specific finite element models were created based on x-ray computed tomography scans. Models were validated by comparison with experimental data. Sensitivity analyses were used to assess the influence of structural parameters such as geometric and material properties. As hypothesized, geometry was found to have a much stronger influence on structural stability than material properties. This information reinforces the notion that deficiencies in tissue strength could be offset by manipulation of stalk morphology, thus allowing the creation of stalks which are both resilient and digestible.

## Introduction

As plant stems grow, their biological processes must simultaneously balance many different factors^1,2^. These functions are balanced based on their perceived need, leading to plant stem systems that grow and develop in response to external abiotic stimuli^3^. If plant stems do not properly acclimate to their wind-loading environment, wind-induced bending can cause failure^4^.

The problem of wind-induced failure is a particular challenge for the achievement of new dual-purpose crops. Such crop could yield high levels of grain while at the same time possessing stems and leaves that are suitable for biofuel production. The leaves and stems of such crops would have low levels of lignin since lignin is an obstacle to biofuel conversion. But lignin deficiencies also cause structural deficiencies, thus causing these plants to be susceptible to wind-induced failure.

**Figure 1:**
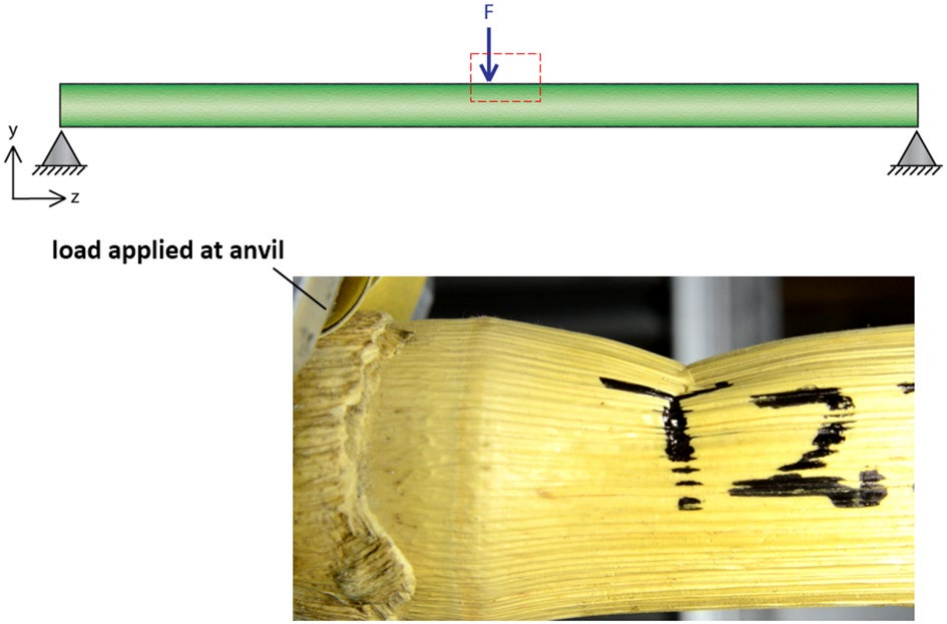
**(**Top) Stalks were tested in three-point bending. (Bottom): Stalks failed due to localized (Brazier) buckling in the area of the stalk immediately above the loaded node.

The primary failure mode of maize is Brazier buckling above the meristem^5^. Brazier buckling is caused by localized instabilities and should not be confused with the more widely-known Euler buckling, which occurs due to longitudinal compression. Brazier buckling occurs when bending stresses cause ovalization of the specimen cross-section. At a critical level of ovalization, structural instability is induced which causes structural collapse^6,7^. Brazier buckling can be induced in laboratory 3-point bending tests^8^. Figure 1 illustrates the loading scheme and a typical failure pattern.

In a previous paper published by our research group^5^, computer simulations were used to explore the relationship between stalk morphology and bending stresses in maize. In these simulations, mechanical stresses were quite sensitive to morphology, but much less sensitive to tissue properties. If this were true, structural deficiencies caused by a reduction in lignin content might be effectively offset by compensatory changes to stalk morphology, thus providing a means for achieving dual-purpose crops.

While this new approach appears to have promise^5^, was exploratory in nature and was based entirely upon computer simulations—without any direct experimental validation. Moreover, that study had several limitations: (1) the simulations examined only bending stresses (not stalk failure), (2) the models used in that study did not allow manipulation of individual morphological parameters, (3) no experimental data was used, and (4) Brazier buckling was not investigated. In fact, to the best of the authors’ knowledge, no previous research has been published on the morphological and material properties that affect the Brazier buckling of maize stems. As a result, the outcome of that paper was a *hypothesis*, not a scientific conclusion. Indeed, the authors acknowledged that further experimentation would be required to fully explore the mechanisms of stalk strength.

The purpose of this study was to test the ideas put forth in the von Forell paper using a combination of experimental data and in silico experiments. This paper is a significant advance upon the results of the previous study for three reasons. First, the computer models used in this study represent a significant improvement upon those of the prior study by (a) capturing a broader range of physical variation, (b) simulating buckling failure rather than linear bending stress, and (c) providing information on specific morphological features. Second, the results of these models were directly validated against corresponding experiments, thus confirming the accuracy and validity of the simulated results. Third, in this study, sensitivities were assessed using both empirical data *and* results of computer simulations. This provides a second point of validation to confirm the reliability of computational results. Overall, the results of this study show a more refined and accurate assessment of the discrepancies between the structural sensitivities of morphological and material parameters of the maize stalk.

## Material and methods

### Overview

**Figure 2:**
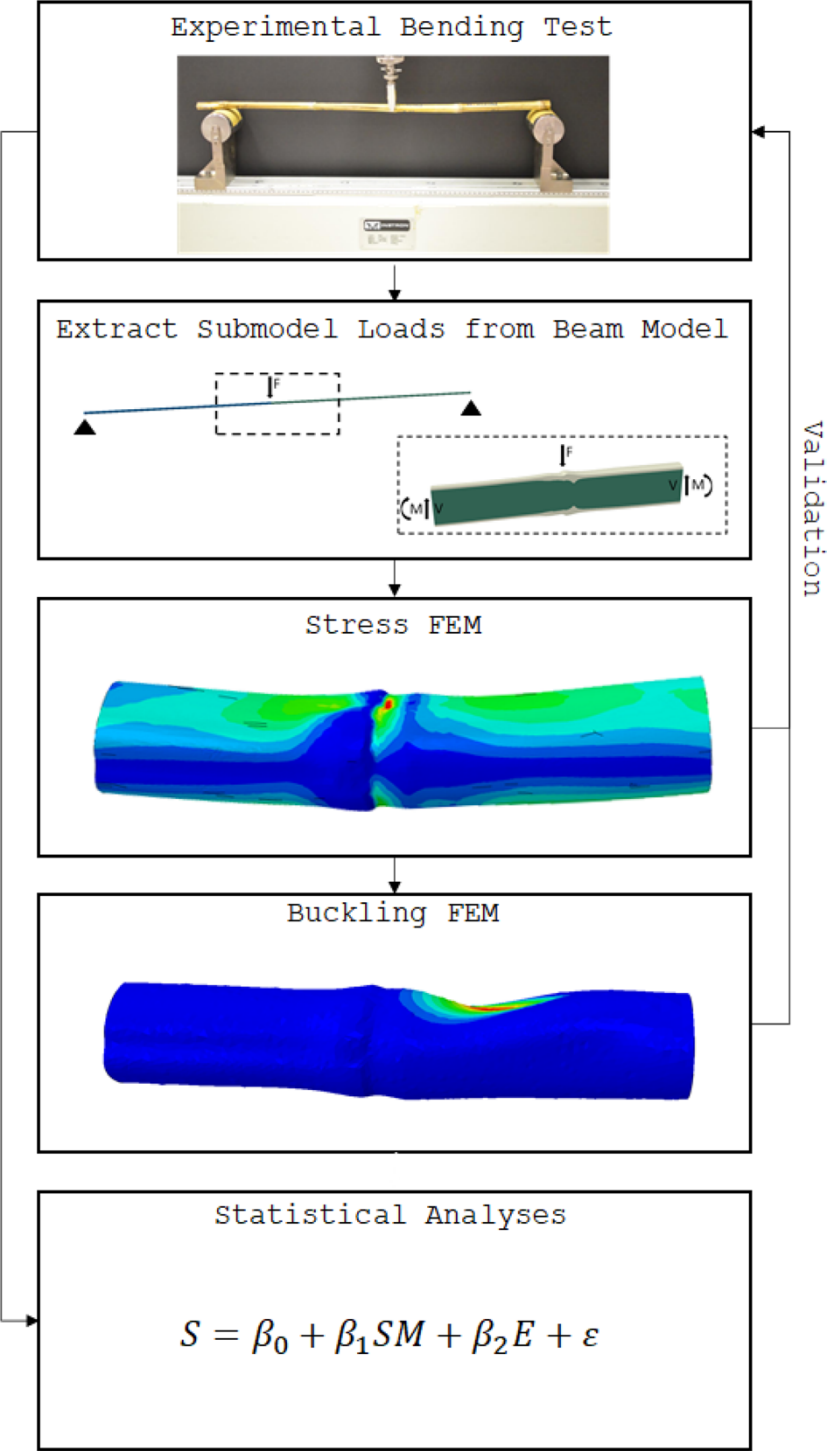
Diagram illustrating the primary components of this study.

This section provides a brief overview of the various components involved in this study, while subsequent section provide detailed descriptions of each component. The study involved three-point bending tests of maize stalk specimens and corresponding specimen-specific in silico finite element simulations. Maize specimens approximately one meter in length with low moisture content were tested in three-point bending according to a previously described testing protocol^9^. Prior to testing, x-ray computed tomography (CT) scans were performed. Specimen-specific finite element models were created from CT scan data (78 micron voxel resolution). The finite element models were validated against experimental data. The finite element models were then used to compute flexural stiffness and critical buckling loads. Material properties were varied to assess their influence on model predictions. Finally, the influence of geometry was evaluated using a statistical analyses of the three-point bending tests in conjunction with geometric data from CT scans. The various elements of the study are shown in Fig. 2.

### Specimens and physical experiments

A previous study from our lab describes the scanning and testing of 980 maize stalk specimens^10^. The experiments and data from that study were used as a starting point for this study. The maize stalks represented 5 commercial dent maize hybrids, and were grown in Iowa at planting densities ranging from 120,000 to 590,000 plants/hectare. Stalks were collected immediately prior to harvest, as this is the most critical period for lodging risk^10^.

Specimens were tested in 3-point bending (see Fig. 2) using the long-span, node-loading protocol described in^8,9^. Force and displacement data were obtained throughout the test, which progressed until stalk failure occurred. The slope of the linear section of the force–displacement curve was used to calculate the flexural stiffness as described in^9^. The maximum force prior to failure, along with the specimen morphology, was used to calculate the failure moment. Results from previous analysis of this data indicated that morphological features in the internodal region of the stalk were most predictive of both flexural stiffness and stalk strength^10^.

### Finite element modeling

#### Geometry and boundary conditions

Maize stalks predominantly fail near a node^5,9^. Similarly, our test protocol^8,9^, produced buckling failure just above the central node. Computational expense was minimized by modeling only the stalk section in the vicinity of the failure region. The geometry of each model was derived from CT scan data. The length of each model was 100 mm, which adequately captured the buckling failure modes while also minimizing Saint–Venant effects^11,12^.

**Figure 3:**
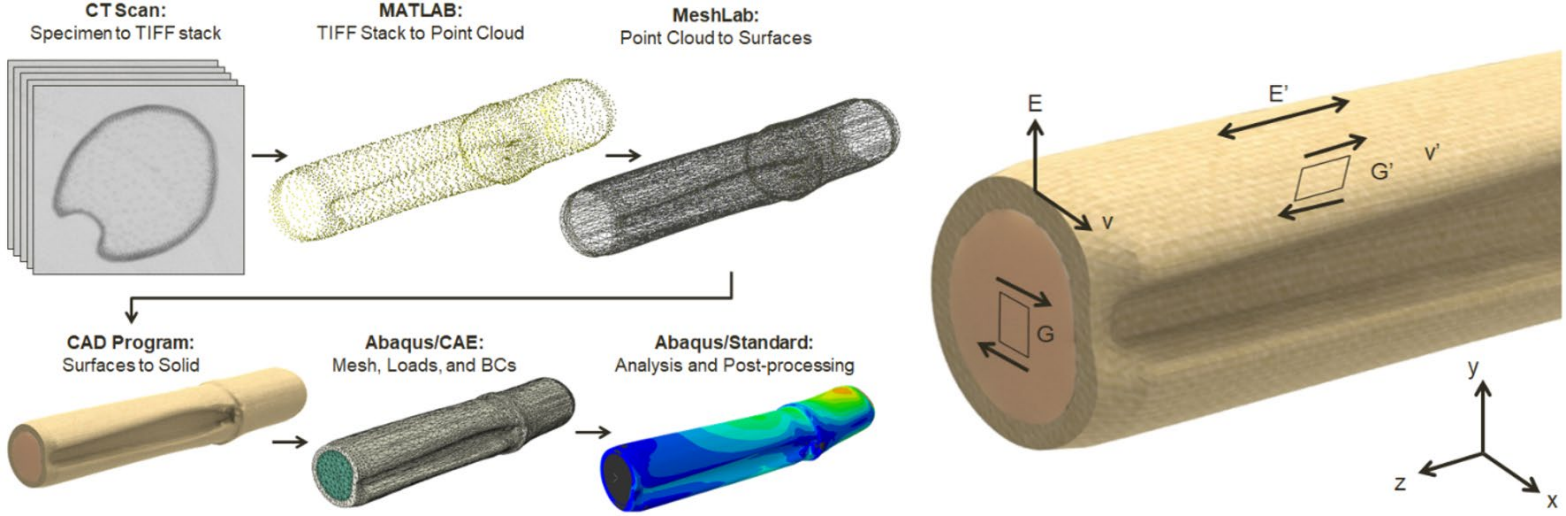
The process used in creating computational models of a maize stem. Left: CT scans were processed to create finite element models using the steps shown. Right: depiction of the rind and pith tissue regions as well as the transversely isotropic mechanical properties.

To create the solid geometry, the CT scans were used to determine both the outer boundary of the specimen and the interface between the rind and the pith^10,13,14^. These boundaries were used to build a 3-dimensional solid specimen-specific finite element model for each specimen. From those boundaries, point clouds were generated and processed in MeshLab 2016–12 to create the outer boundary and pith-rind delineation surfaces using a screened Poisson reconstruction. These surfaces were imported into SolidWorks 2016, and solid models of the rind and pith were generated. The solid models were then migrated into Abaqus/CAE 2016 for constructing and meshing the finite element models. This process is shown in Fig. 3.

Shear and bending loads were applied to the end faces of each finite element model. These loads were calculated from the loading configuration of the 3-point bending experiments (Fig. 2). With loads applied in this manner, the loading anvil was assumed to be immobile. As a result, the point of contact between the anvil and the stalk was fixed in all 6 degrees of freedom. Although this constraint was required for model stability, it also imposed rotational constraints. However, the resultant moments at the point of contact (indicators of the degree of constraint), were found to be negligible, thus indicating that this boundary condition did not adversely influence the validity of the model.

**Figure 4:**
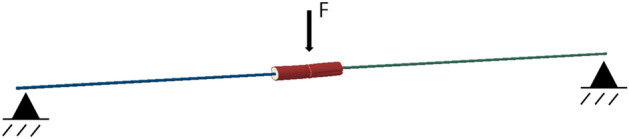
The solid continuum sub-model computationally stitched in to a three-point bending beam model.

To validate the geometry used in the model, the sensitivity models were stitched in to global specimen beam models, referred to herein as continuum-beam models, and compared to three-point bending experiments of the stem specimens. A beam model was constructed to replicate the three-point bending tests for six of the specimens. Each model consisted of a single beam instance with the longitudinal Young's modulus and second moment of area derived from the experiment. The specimen-specific sensitivity model was then stitched into the beam model and compared to the experiment. The continuum-beam model is shown in Fig. 4.

#### Software, mesh, and solver routines

All finite element models were developed in Abaqus/CAE 2016. The geometry was meshed using 10-noded quadratic tetrahedral elements^1^. The flexural stiffness analyses used a linear, full-Newton direct solver. The buckling analyses used a subspace eigensolver.

#### Material properties

The corn stalk was modeled as having two distinct materials: rind and pith. Both materials were modeled as transversely isotropic to represent the longitudinal fibers present in both tissues. Five independent tissue properties are required for such materials, but many of these tissue properties have not previously been measured for maize tissues. In this study, values for the longitudinal modulus of the rind were based on experiments previously published for these same specimens^15,16^.

The remaining material properties were calculated based on ratios derived from prior research, tests performed in our lab, and estimation. The material properties of the pith were estimated as 1/100th of those of the rind based on unpublished experiments performed in our lab. These estimation rules were used only to obtain initial values for each parameter. As described in the following section, parametric analysis was used to independently vary these material properties for each specimen-specific in silico experiment. Prior sensitivity studies from our research group^5,14^ showed that the longitudinal and transverse Poisson’s ratios (ν′, ν) of both the rind and the pith had an extremely small effect on model behavior. As a result, these parameters were not varied in this study. The material properties used in this study are summarized in Table 1.

**Table 1:** Mechanical properties ratios of maize stalk tissues reported/used in previous experimental and modeling studies.

Ratio and rind longitudinal elastic modulus to material property		Previously reported data			This paper	10X ratios
		Maize	Softwood, extrapolated for pith**	Von Forell paper***		
Rind	E′	1	1	1	1	1
	E	15	10	10	100	10
	G	–	109	250	1000	100
	G′	–	12	10	10	1
	E′	200	–	15	100	100
	E	500	–	15	10,000	1000
	G	–	–	375	100,000	10,000
	G′	–	–	250	1000	100

Since some maize properties have never been reported in the literature, typical mechanical properties of softwood species are also provided as a point of reference. Note that all properties are reported as ratios of the Young’s Modulus of the rind (top row). Ratios are also shown for an order of magnitude change between E′, E, G′, and G, shown in the rightmost column.

*^14,16^, **, ***^5^.

As many material properties are still unknown in maize, these estimates—and the estimates of the previous study^5^ may be incorrect. It is certainly true that the choices of material properties will influence the absolute values for structural responses. However, since the models used in this study are linear, and because our analysis focused on sensitivity analyses, the choice of material properties had a negligible effect on normalized sensitivity values. This is because, mechanical material properties have a linear influence effect on the system behavior. Thus the normalized sensitivities (which are non-dimensionalized partial first derivatives) are virtually independent of the material property values themselves. To confirm this, additional cases were analyzed with the ratio between E′, E, G′ and G modified by an order of magnitude (see “10X Ratios” in Table 1). The normalized sensitivity values were compared to the baseline ratios in this study for both linear buckling and flexural stiffness.

### Sensitivity analyses

Simulations produced estimates of flexural stiffness and stalk strength (critical buckling failure load) for each specimen-specific finite element model. Sensitivity analyses were performed by computing the relative change resulting from small changes in material or geometric parameters. Normalized sensitivity values were computed as the normalized difference in input divided by the normalized difference in output^5^:

1 $$S = \frac{{\left( {Y_{new} - Y_{ref} } \right)/Y_{ref} }}{{\left( {X_{new} - X_{ref} } \right)/X_{Rf} }}$$

where *Y*_*ref*_ and *Y*_*new*_ indicate the finite element response at the reference and modified (new) configurations, while *X*_*ref*_ and *X*_*new*_ represent the reference and modified values of the varied parameter. For example, to obtain the sensitivities to material properties each model was first analyzed with its own baseline material properties (the reference case). Next, new models were created for each of the 8 material properties (E′, E, G′, and G for both rind and pith). These new models were identical to the original model, but one material property in each model was increased by 1%. Each model was then analyzed to obtain the 8 corresponding *Y*_*new*_ values. In addition, to determine the total effect of the pith, four specimens were analyzed with the pith tissue entirely removed from the models.

#### Geometric sensitivities

The complex geometry of the maize stalk presents a challenge for a parametric sensitivity analysis since CT scan data of the maize stalk geometry is essentially a spatial point-cloud with thousands of individual points. None of these points are individually meaningful, and geometric features of point could data are not easily modified. Two approaches were therefore used to assess geometric sensitivities: a statistical approach (described later), and a geometric sensitivity based on *carefully chosen pairs of maize specimens*. In this latter approach, data mining of the 980 CT scans was used to identify specimens pairs that exhibited negligible differences (< 1%) in two of the primary geometric parameters (major diameter, minor diameter, and rind thickness), but a significant difference (more than 7%) in the remaining parameter.

Geometric parameters obviously vary along the length of each stalk. The comparisons described above were made for each stalk in the internodal region. This region was chosen for two reasons. First, there is virtually no longitudinal variation of morphology in this region, which eliminates subjectivity. Second, this region has previously been found to be highly predictive of stalk strength^10^. The selection process described above yielded one pair of stalks for each of three geometric parameters, thus providing a sample size of 6 specimen-specific finite-element models. For example, the influence of rind thickness was assessed by identifying two stalks that had very similar major and minor diameters, but for which there was a substantial difference in rind thickness. The sensitivity of the stalk to rind thickness was then calculated as described in Eq. (1). To control for potential material interaction effects, all of these models were assigned identical material properties.

#### Parametric design

For the buckling study, 12 specimen-specific finite element models were analyzed for each of the 9 material variation cases. For the flexural stiffness study, 11 specimen-specific finite element models were analyzed for the 9 material variation cases. In addition, 6 specimen-specific finite element models were analyzed for the complete removal of the pith in linear buckling. This resulted in a total of 213 finite element analyses.

### Empirical sensitivity analyses

Empirical test data was used to provide further insight into geometric sensitivity. Empirical sensitivity analyses were performed on the three-point bending data, which consisted of three predictor variables: major diameter, minor diameter, rind thickness, and longitudinal Young’s Modulus of the rind, as well as two response variables: flexural stiffness and maximum stalk strength.

Non-dimensional sensitivity values can be obtained via a multiple regression approach as described by^18^. The challenge we encountered in applying this method was severe collinearity between the minor and major diameters of the stalk (R^2^ = 0.92). Under such circumstances, regression coefficients should not be interpreted as sensitivities^19^.

We therefore used a slightly different approach in this study. In a previous study, we used mechanics-based regression to identify a very high linear correlation between the section modulus of the stalk and stalk strength^10^. In that same study, we also reported that the cross-section of the maize stalk can be approximated by an ellipse with a remarkable degree of precision. In fact, the maize stalk is so well approximated by an ellipse that the correlation between the section modulus as measured from CT data and the section modulus as obtained when assuming that the stalk is a simple ellipse exhibits an R^2^ value of 0.96. We therefore performed a regression in which section modulus and Young’s Modulus were used to predict stalk strength (a similar analysis was used to predict flexural stiffness). These regression equations are provided here:

2 $$S = \beta_{0} + \beta_{1} SM + \beta_{2} E + \varepsilon$$

3 $$FR = \beta_{0} + \beta_{1} I + \varepsilon$$

where *S*, *SM*, *E*, *FR*, and *I* represent Strength, Section Modulus, Young’s Modulus, Flexural Rigidity, and Area Moment of Inertia, respectively. Strength and flexural rigidity were obtained from physical tests while section modulus and moment of intertia were obtained through numerical analysis of CT scans[details in^10^. Regression was then performed to obtain the *β*_*i*_ values.

After *β*_*i*_ values were obtained, the Section Modulus and Moment of Inertia were then assumed to be dependent on the major diameter, minor diameter, and rind thickness (*a*, *b,* and *t*) of an elliptical cross-section. Under this assumption, these quantities can be expressed as follows:

4 $$I = \frac{\pi }{4}\left( {ba^{3} - \left( {b - t} \right)\left( {a - t} \right)^{3} } \right)$$

5 $$SM = \frac{\pi }{4}\left( {ba^{3} - \left( {b - t} \right)\left( {a - t} \right)^{3} } \right)/a$$

With *β*_*i*_ values known, Eq. (4) was substituted into Eq. (3), and Eq. (5) was substituted into Eq. (2). This produced the following equations for strength and flexural stiffness as functions of *a*, *b,* and *t:*

6 $$S = \beta_{0} + \beta_{1} \frac{\pi }{4}\left( {ba^{3} - \left( {b - t} \right)\left( {a - t} \right)^{3} } \right)/a + \beta_{2} E$$

7 $$FR = \beta_{0} + \beta_{1} \frac{\pi }{4}\left( {ba^{3} - \left( {b - t} \right)\left( {a - t} \right)^{3} } \right)$$

These equations were *analytically* differentiated to obtain sensitivity equations for *FR* and *S* as functions of *a*, *b*, and t. Finally, specific values for *a* (major diameter), *b* (minor diameter)*,* and *t* (rind thickness) were obtained from CT data and entered into each sensitivity equation. This produced empirical sensitivity values for strength and flexural stiffness as influenced by major diameter, minor diameter, and rind thickness. The empirical sensitivity analysis was based on 956 experimental tests and therefore produced 956 sensitivity values.

### Use of plant materials

Studies complies with local and national regulations.

## Results

### Validation

The response of specimen-specific models closely matched the behavior of their physical counterparts. The correlation between physical and computational values of flexural stiffness was very high (*R*^*2*^ = 0.99), and the flexural stiffness predictions were quantitatively accurate. The close agreement between the experimental stiffness of each specimen and the computational stiffness of each specimen provides validation that this modeling approach is appropriate.

**Figure 5:**
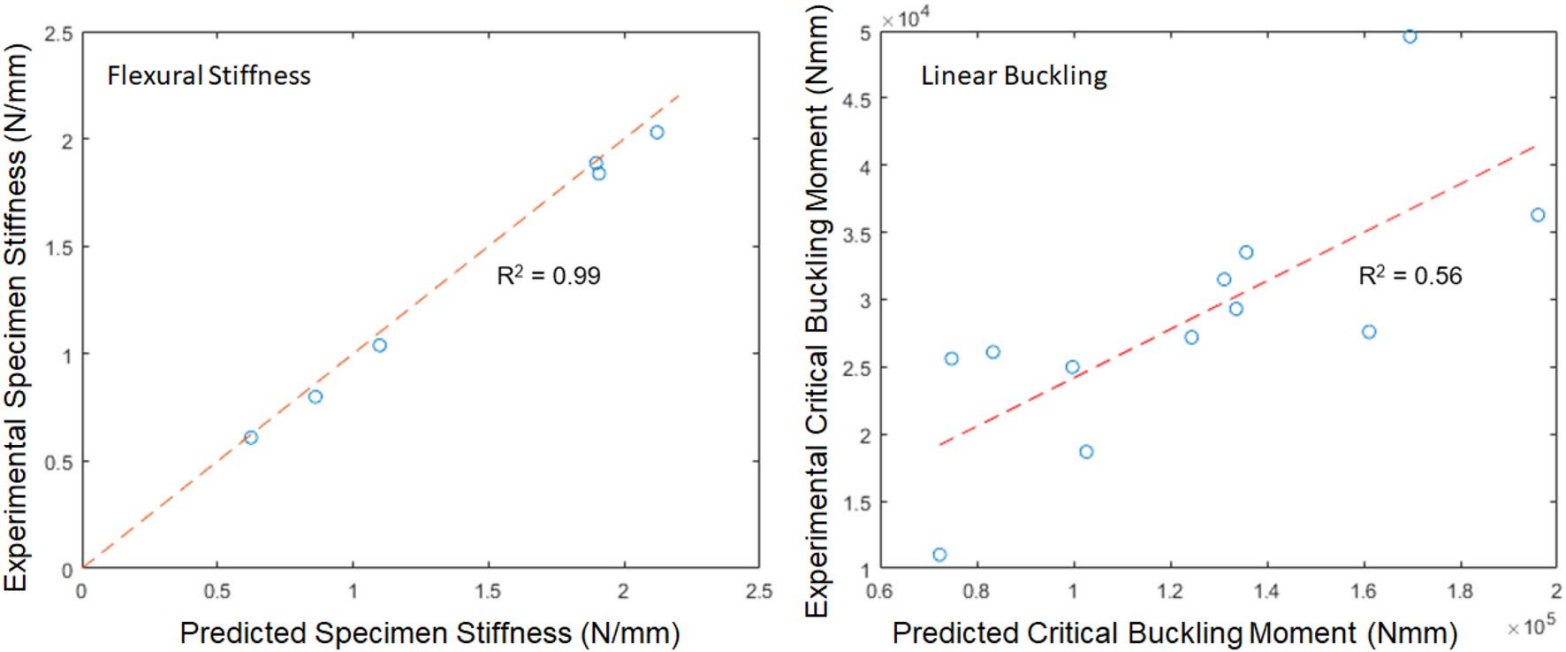
Model validation correlation plots, consisting of comparisons between predicted and measured values of flexural stiffness (left), and buckling load (right).

Next, the stalk strength obtained from simulated buckling was compared to the stalk strength of the experimental three-point bending specimens. Computational buckling analyses almost always over-predict actual buckling loads. The reason is that the linear buckling analysis does not account for nonlinear factors such as cross-sectional ovalization, random points of tissue irregularity, and tissue failure itself. Thus, as expected, the simulated stalk strength values were higher than the actual loads at failure. But the *R*^*2*^ value of 0.56 between measured and predicted buckling load indicates that the model captures the main effects of buckling (with the understanding that linear buckling always overestimates critical buckling loads). These results are shown in Fig. 5.

### Computational sensitivity results

**Figure 6:**
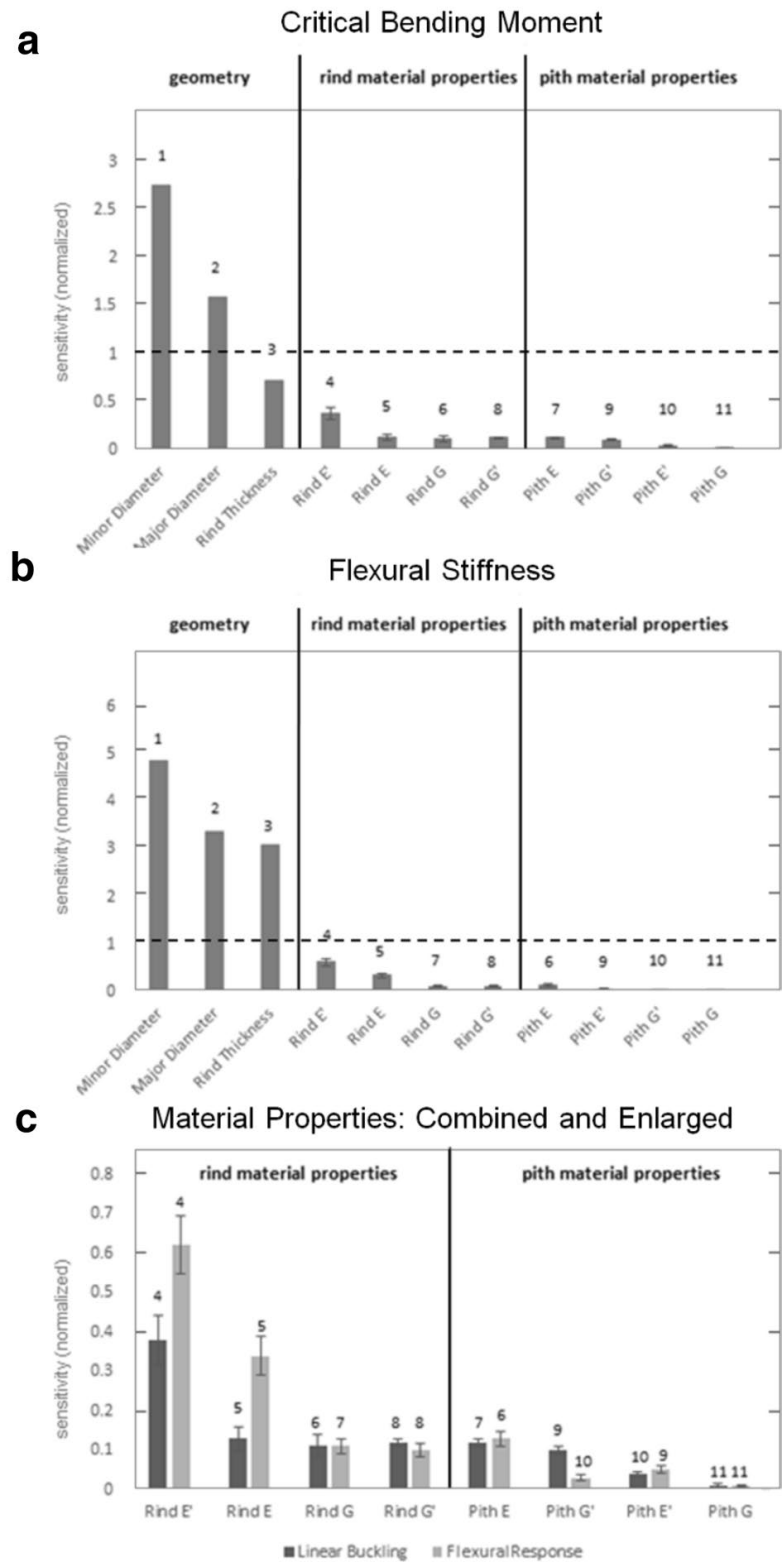
Normalized sensitivities (% change in output / % change in parameter) of geometric and material parameters. (**a**)—linear buckling (**b**)—flexural stiffness. Panel (**c**) contains both results, and is enlarged to show the results of material parameters only. Whiskers represent 95% confidence intervals on the means. The numeral above each bar indicates that parameter’s ordinal rank.

Sensitivity analyses of computational models were performed to determine the effect of each geometric and material parameter on stalk strength and flexural stiffness. The mean sensitivities for each parameter are shown in Fig. 6. Geometric parameters were consistently found to be more influential than any of the material parameters for both analyses. The rind tissue properties generally had a larger impact on flexural stiffness than the pith material parameters. Of the geometric parameters, minor diameter was the most significant, major diameter was the second most significant, and rind thickness was the least significant.

The longitudinal Young’s modulus of the rind was the most significant material property for both analyses. The rank ordering of the material parameters differs slightly between the flexural stiffness and buckling studies, as is shown in Fig. 6. In addition, removing the pith entirely reduced the critical linear buckling load by an average of 25.5% (standard deviation of 2.9%).

### Empirical sensitivity results

**Figure 7:**
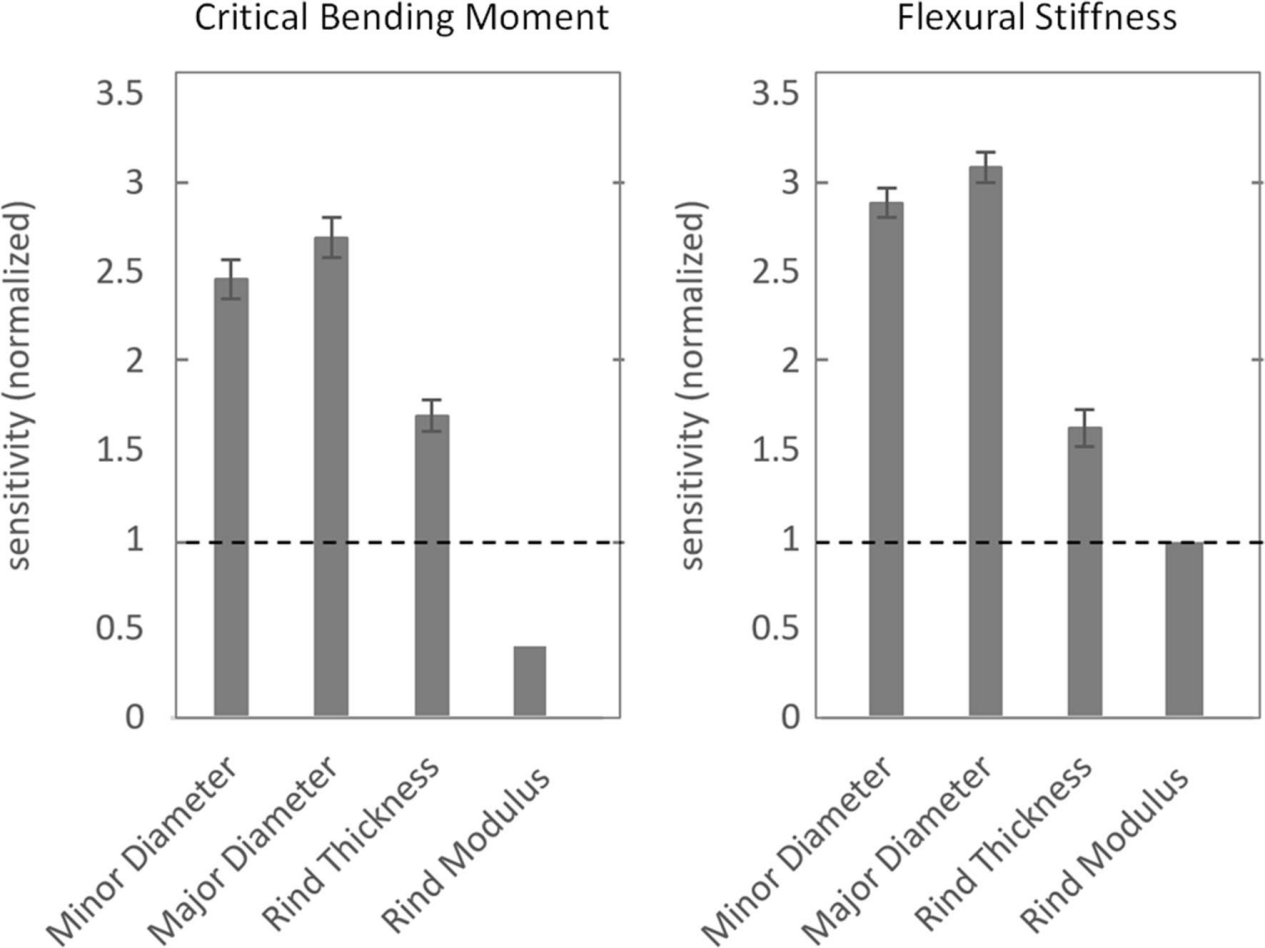
Empirical normalized sensitivity values Normalized univariate regression (n = 956) coefficients of geometric parameters for critical bending moment (left) and flexural stiffness (right), with 95% confidence intervals on the regression coefficients, compared with finite element geometric sensitivities.

The baseline regressions (Eqs. 2 and 3) exhibited R^2^ values of 0.80 and 0.78, respectively. Major and minor diameters were found to be the most influential parameters for both flexural stiffness and stalk strength. Rind thickness was the least influential geometric parameter for both analyses. The longitudinal Young’s Modulus of the rind was found to have a sensitivity of 0.43 and 1.0 for strength and stiffness, respectively. The mean sensitivities for this analysis are shown in Fig. 7.

### Combined results

**Figure 8:**
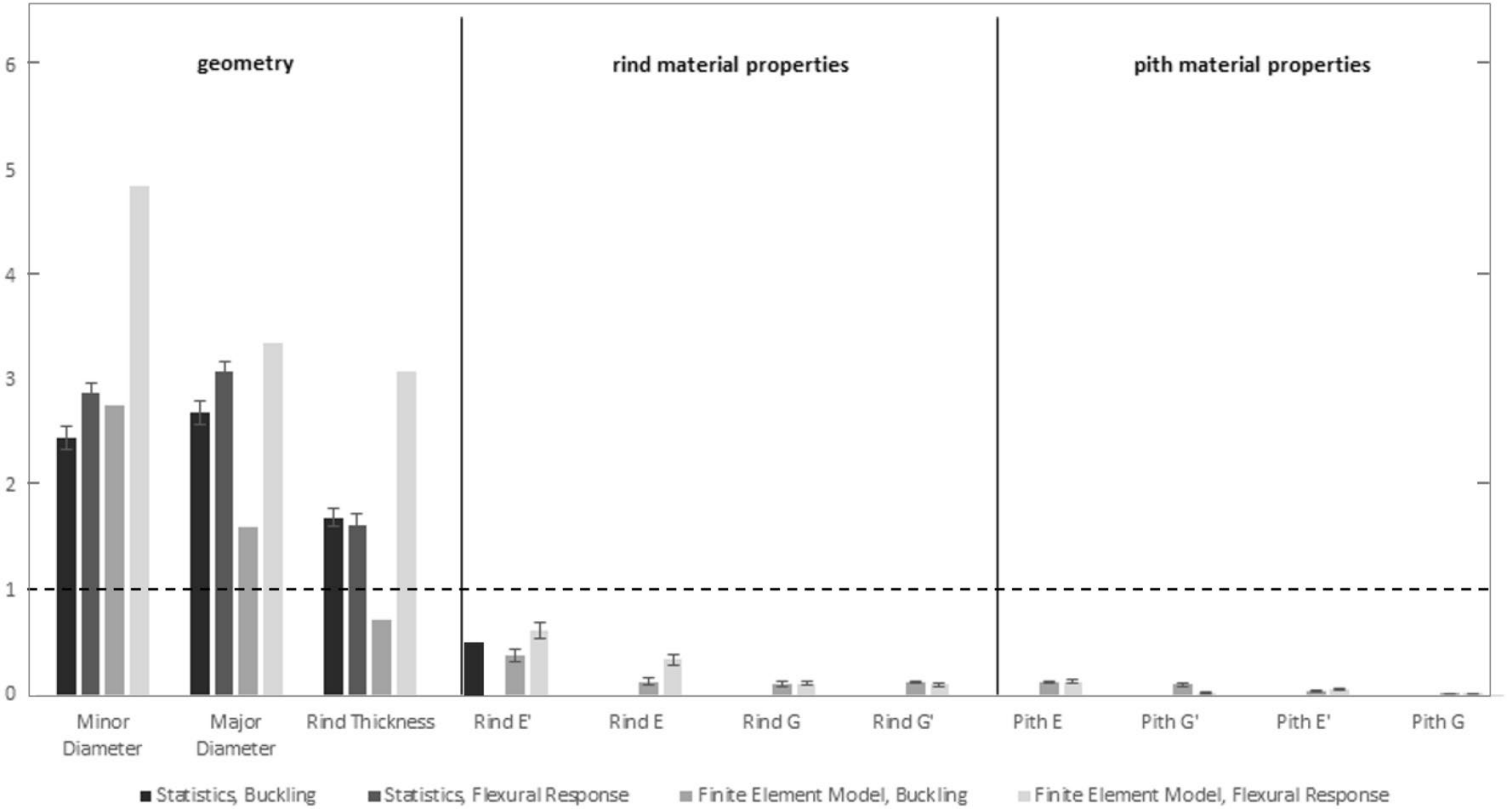
All sensitivities from Figs. 6 and 7 on a single plot for purposes of comparison. Empirical sensitivities are shown in dark gray shades while computational sensitivities are shown in lighter gray shades.

All results were combined into a single figure (Fig. 8) to illustrate the sensitivity of maize stalk parameters to the parameters varied in this study. This figure clearly indicates the dominant effect of geometric parameters over those of the rind and pith.

### The influence of material property estimates

As mentioned previously, many material properties of the maize stalk are not yet known. As such, it was necessary to use estimates in computational models for several of the material properties used in this study. However, because of the linear nature of these models, and the fact that normalized sensitivities depend upon *changes* in response rather than the magnitude of response, we anticipated that the material properties would have minimal influence on the system sensitivities. To confirm that this was the case, the ratios between E′, E, G′ and G were modified by an order of magnitude (see “10X Ratios” in Table 1). The resulting sensitivity values were (on average) within 3% of the results reported above. These results indicate that our material property estimates had very little influence on the normalized sensitivity values reported above.

## Discussion

In the broader scientific discussion on lodging, chemical composition is often mentioned, but morphology and mechanical tissue properties are not commonly addressed. Yet from a structural engineering perspective, these factors are far more relevant and influential than material properties when predicting structural response such as stiffness, strength, etc.. This study demonstrates through both empirical and computational approaches that geometric factors have a strong influence on bending stiffness and stalk strength, with material properties having a much lower influence.

A 2015 study from our research group^5^ obtained similar *rankings* as those presented above. However, the results in that study showed much more pronounced discrepancies between the sensitivities of geometric and material properties. The results in this study are considered to be more accurate for several reasons. First, the models used in this study were thoroughly validated against experimental data (the models in the previous study were not). Second, this study used a broader sample of model geometries and used geometric factors that were more representative of the actual maize morphology (the previous study used limited geometric sampling). Thirdly, this study used a transversely isotropic material model for the pith tissue (as opposed to the isotropic model used in the prior study). Finally, and perhaps most importantly, the structural response of interest in^5^ was maximum bending stress, not stalk strength or flexural stiffness, both of which are closely correlated with physical bending strength^20^.

This study should also be placed in context with a purely empirical study with similar conclusions^10^. That study also reported that morphological parameters were strong predictors of stalk strength (as quantified using statistical analysis). There are two major differences between this study and^10^. First, that study examined broad-based predictive power across specimens, not local sensitivity within individual specimens. Second (and more importantly), that study did not provide any insight on the influence of material properties. In contrast, this study provided direct comparisons between the influence of both categories of factors.

Other studies have also reported that factors such as dry weight per unit length are good predictors of lodging resistance^21,22^. This metric may be a of some practical use since measurements of linear density could conceivably be automated quite easily. However, the problem with this metric is that linear density depends upon both geometry and material properties (primarily tissue density). This approach confounds geometric and material properties into a single measurement, which obscure the influences of individual factors.

The use of computational models in this type of study must be undertaken with great care and attention. These models are so convenient and realistic, that there is sometimes a tendency to over-interpret their results. The primary advantage of computational models is the ability to manipulate the system of interest in ways that are not possible using an experimental approach. The drawback to computational models is that their results should almost always be interpreted as suggesting possible hypotheses rather than providing confirmation of a hypothesis. This is because computational models are (by their very nature) *approximations* of reality. As a case in point, both the von Forell et al. paper and the current study identified geometric parameters as being more influential than material parameters. However, the discrepancy between geometric and material parameters was exaggerated in the prior study, which estimated the discrepancy as having a difference of 18 times. The empirical data presented in this study indicates a much more moderate difference of approximately 3.3 times. This is because material parameters were found to exhibit a larger influence on stiffness and strength (this study) than on linear elastic maximum stress (previous study).

This study provides further support for the generalization that the strength of maize stalks is primarily determined by morphological features, with mechanical tissue properties having a relatively weak influence on stalk strength. However, more work will be required to understand the specific modes and mechanisms of failure in maize stems to better understand which continuum mechanics theories best predict the behavior and failure of the system. In particular, more work needs to be done to quantify how these relationships change with irregular morphologies and the details of how the pith influences stalk strength.

### Limitations of this study and future work

This study relied upon geometrically realistic, but relatively simple models. Finite element models did not include non-linear effects such as large deformations, tissue fracture, or tissue collapse. The materials used in this study were all modeled as linear elastic. This study did not investigate the influence of tissue *strength* on buckling as, at this time, there are no known studies that have reported the compression strength of the maize tissues.

The estimation of geometric sensitivities using computational models of specially chosen specimen pairs, while novel in approach, did not produce results that were consistent with the other methods used in this study. This is likely due to the fact that the criteria for “matching” specimens was relatively simplistic in comparison to the full geometric complexity of the maize stalk. The failure of this method to produce useful information highlights the need for parameterized models of plant stems that could be used to perform sensitivity analyses though the controlled modification of stalk geometry. Such models will enable many additional studies such as structural optimization, etc.

In spite of these limitations, the major trends observed via each method were similar in nature. The empirical sensitivity results were based on an extensive set of tests and produced results that were generally aligned with the results of model-based sensitivity results. More advanced models and experiments will be necessary to provide further insights into issues such as the onset of buckling, and the role of tissue failure. But the trends shown above are unlikely to be affected in a major way.

In performing parametric sensitivity analyses, significant mechanistic insight into the system can be achieved. This method allows us to rank order each parameter by its individual effect on each complex phenotype or failure mode of the system, a task that is often impractical through experimentation of specimens with unknown material properties and complex geometries. It should be noted, however, that this approach is limited by the fact that these hypotheses drive towards a mechanistic understanding of each parameter individually. Although helpful, this ignores complex relationships that likely exist between parameters. For example, the material properties and geometry of a maize stem may be influenced by biomass allocation trade-offs, biotic influences, and abiotic factors throughout the plant’s lifespan, and by the interactions of those factors with the constantly-changing plant itself. While we have shown above that the strength and flexibility of the corn stalk are most sensitive to changes in morphology, future work should consider interactions between material and morphology. For example, this analysis does not consider the increased biomass loads that could result from a change in morphology, and this analysis does not address the possibility that morphology and material properties may be intrinsically linked. Ultimately, a marriage of the individual influences of the parameters with the interrelationship between those parameters and their environmental context is necessary in building a complete understanding of the system.

## Conclusions

Using a combination of validated models and empirical data, this study conclusively demonstrated that the morphology of the maize stalk has a much greater influence on structural flexibility and strength than mechanical tissue properties. However, difference in morphological and material sensitivities was found to be significantly lower than previously estimated^5^. Major and minor diameters were found to be the most influential parameters, followed by the rind thickness. Of the material properties examined in this study, the longitudinal Young’s modulus of the rind was found to be the most influential, but material properties generally exhibited a weak influence on structural response. The dominance of geometric parameters was supported in this study by both empirical data and results from validated computational models. This conclusion is consistent with other studies in the literature, though most studies indirectly address the difference in sensitivity between these parameter categories^5–7,10,12,20,23^.

This study provides clear evidence in support of the idea that deficiencies resulting from targeted reductions in organic polymers (such as lignin) may be counterbalanced by adjusting stalk morphology. Drawing on state-of-the-art techniques from two disciplines, plant scientists and engineers could work together to *design* a crop stalk architecture that would enable simultaneous first/second generation biofuel production: crops that are high yielding, structurally robust, and whose biomass is readily converted to biofuel.

## Data Availability

The data sets used and/or analyzed within the current study are available from the corresponding author on reasonable request and with approval of the research sponsor (Bayer AG).
